# Study on the fastening characteristics of preformed helical fittings considering the surface effect of transmission lines

**DOI:** 10.1038/s41598-023-36982-9

**Published:** 2023-07-06

**Authors:** Lv Zhongbin, Liu Guanghui, Li Fangyu, Wu Chuan, Tao Yaguang, Liu Jufang, Ye Zhongfei, Liu Xiaohui, Sun Yuntao, Yan Bo

**Affiliations:** 1 College of Aerospace Engineering, Chongqing University, Chongqing, 400044 China; 2 State Grid Henan Electric Power Research Institute, Zhengzhou, 450052 China; 3 School of Civil Engineering, Chongqing Jiaotong University, Chongqing, 400074 China; 4 BYD Co., Ltd, Shenzhen, 518118 China; 5 State Key Laboratory of Mountain Bridge and Tunnel Engineering, Chongqing Jiaotong University, Chongqing, 400074 China; 6 Nanjing Electric Power Fittings Design & Research Institute Co., Ltd, Nanjing, 210009 China

**Keywords:** Electrical and electronic engineering, Applied physics

## Abstract

Because of their superior mechanical properties, preformed helical fittings are widely used in UHV transmission lines. However, they easily slip and become loose under extreme environments, so it is very important to study the fastening characteristics of preformed helical fittings. According to the stress characteristics of preformed helical fittings, a parametric finite element model including a core and preformed armor rods was established. Finally, the finite element model calculation was verified by comparing it with the test results. In this paper, the influences of the preformed armor rod diameter, pitch, length and forming aperture on the fastening characteristics were investigated. The numerical simulation results showed that the smaller the forming aperture of preformed armor rods, the larger the grip force. However, a small forming aperture is inconvenient to install, and too large of a grip force on the core easily leads to core damage. With the increase in the preformed armor rod length, the grip force increased gradually and linearly, and the increase slowed after the pitch number reached 9. The larger the pitch, the smaller the grip force of the preformed helical fittings. The fastening characteristics of preformed armor rods with slightly larger diameters were better and the fourth power of the diameter of the preformed armor rods has a linear relationship with the grip force.

## Introduction

**Figure 1:**
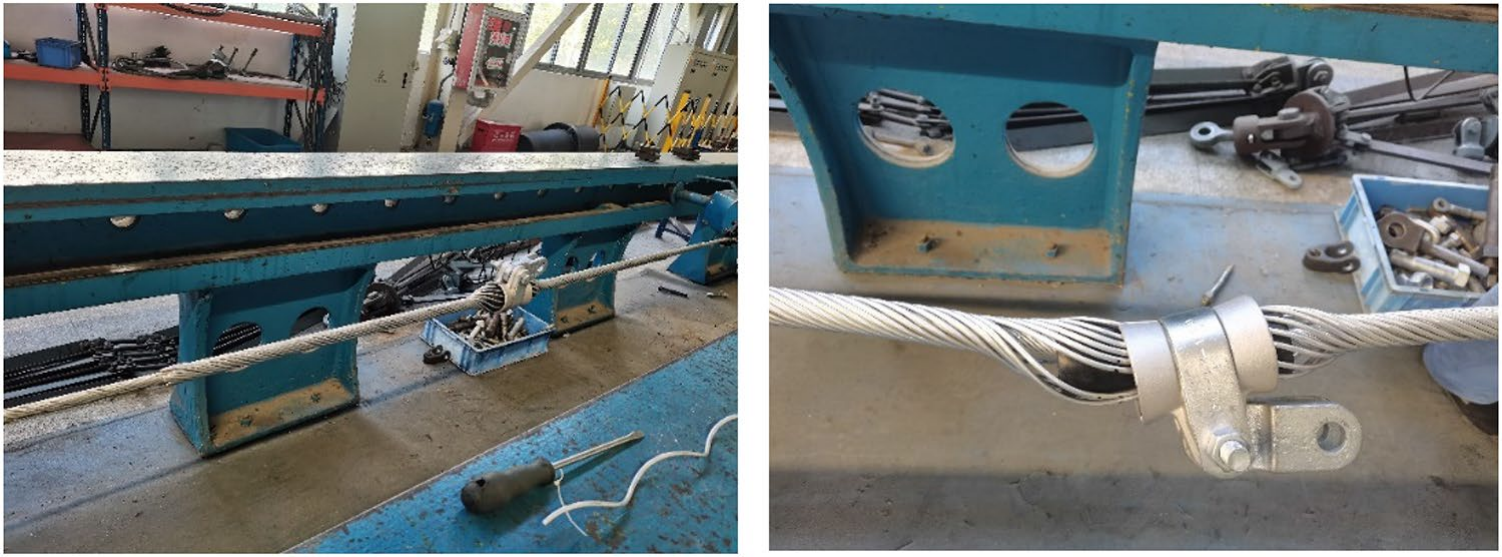
The normal working state and failure state of preformed helical fittings.

With the implementation of the “West Power East Transmission” project in China, the use of 750- and 1150-kV ultra-high voltage (UHV) lines will become more and more extensive. The extensive use of preformed armor rod clips makes them more likely to fail. Based on the overall statistics of the faults of ground wire metal tools of the existing projects in Xiangshang, Jinsu, Xizhe, Hazheng, Lingshao, and Jiuhu, 57 ground wire faults have occurred in total, of which 37 were ground-wire preformed armor rod clip faults. Photographs of failing preformed helical fittings are shown in Fig. 1.

Preformed helical fittings have been successfully used in many high-voltage line projects around the world, and the application of preformed helical fittings in China was relatively late. There have been few research results on the fastening characteristics of preformed helical fittings. Because the steel strand and preformed helical fittings have similar geometric structures, the preformed helical fittings can be analyzed by referring to the research results of steel strands^1–3^.

Finite element analysis is an important method for studying steel strands. Given the establishment of steel strand and preformed armor rod models, Stanova et al.^4,5^ (2011) have established mathematical geometric models of single strands, double strands, and spiral triangular strands, determined the centerline equations for S- and Z-twist strands and any circular steel wires and derived and presented specific forms of the parametric equations. Judge et al.^6^ developed a fully three-dimensional (3D) elastoplastic finite element model of a multilayer spiral stranded cable. They designed a new program that could generate complex geometries and finite element meshes. These models can predict the local and global responses of cables to failure, and they are consistent with experimental data. Thus, they have great application prospects.

A mesoscale mechanical model of the bending behavior of helically wrapped cables under tension was developed by Hong et al.^7^ The model accounts for the nonlinear dissipative behavior of the cable arising from the slippage of wires under friction forces. It was shown that the bending stiffness rapidly diminished with increasing cable curvature over a range that can be as large as two orders of magnitude. Meng et al.^8^ used a new semi-analytical method to study the inter-wire contact effect on the mechanical performances of a wire rope strand. They established a mathematical model of the wire rope strand subjected to axial tensile forces and torsional loads and solved the model equations to study the mechanical properties of the wire rope. In addition, they used the conjugate gradient method and the fast Fourier transform to calculate the deformation, contact pressure, and internal stress of the wire contact point caused by the contact between the wires.

Jolicoeur^9^ and others presented a semi-continuous model of multilayer strands under bending, tensile, and torsional loads. It was based on continuum mechanics and the elasticity of orthotropic materials. The model permitted the evaluation of the strand stiffness, contact stress, interlayer shear stress, and interlayer slip.

Because preformed armor rods and steel strands have significant structural similarities, it is feasible to study preformed armor rods with reference to the research methods for steel strands. Ghoreishis et al.^10,11^ found that in the field of cable modeling, many models have been proposed to describe the mechanical behavior of simple straight strands under axial loads, and they compared the predictions of these models with existing experimental data. However, due to limited trial results, the effectiveness of these models was not evaluated. Therefore, to verify the validity of the strand analysis models, they studied the elastic behavior of a simple 6 + 1 strand under axial static loads, and the results showed that the calculation results of this three-dimensional finite element model were very close to the existing test data. Thus, these results can be used as a reference for the mechanical properties of the cable.

Nawrocki et al.^12,13^ proposed a finite element model of linear wire strands that could examine possible interline motion. The effects of pure axial loads and contact conditions combining axial loads with bending were studied. Compared with existing experimental and theoretical data, the model proved to be reliable. The interline rotation and sliding controlled the reaction of the cable under axial and bending loads, respectively, and the rotation could be viewed as free. Jiang et al.^14–17^ established a finite element model of three layers of straight spiral wire ropes with axial loads (tensile and torsional forces). The spiral symmetry of the wire rope was used to establish precise boundary conditions while taking into account friction, contact, and plastic yield. In addition, the model allowed the determination of local stress distributions, in particular, the model revealed the inhomogeneity of the stress distribution of the outer spiral caused by the contact of grid points.

Wang et al.^18,19^ performed finite element analysis on lifting wire ropes and three-layer steel wire ropes, and they explored the fretting fatigue parameters and stress distribution cross sections of cable wires. A more realistic three-dimensional modeling approach and finite element analysis of wire ropes were presented by Erdonmez and Imrak^20^. A single helical geometry was sufficient to model a simple straight strand, while an independent wire rope core (IWRC) had a more complex geometry due to the inclusion of double helical wires in the outer strands. Based on double helical wires, three-dimensional IWRC modeling was applied for both right regular lay and lang lay IWRCs. Frigerio et al.^21^ proposed a three-dimensional finite element modeling method whose model focused on analyzing complex deformation mechanisms and nonlinearities associated with spiral wire contact and residual stress states during manufacturing. The results showed that when the residual stresses in the manufacturing process were included in the calculation, the inelastic elongations at load levels that were significantly lower than the nominal yield strength of the constituent wires were within a reasonable range of variations.

Chen et al.^22^ developed a new parametric geometric model of a spiral triangular stranded line in which the two strands exhibited elastoplastic behaviors under both axial tensile and torsional loads. Fontanari et al.^23^ and others studied the elastic plasticity response under axial load conditions that exceeded the elastic limit. Ma et al.^24^ applied differential geometry theory and the ANSYS software to perform geometric and finite element modeling of 6 × 19IWS wire ropes. Tsai and Mall^25^ performed elastic plasticity analysis of the fretting stresses in a prestressed strip in contact with cylindrical liners using the finite element solver ABAQUS.

There are also many research results on the preformed helical fittings used in China. Wu et al.^26,27^ established a structural field simulation model of the contact point between a preformed armor rod and a ground wire and analyzed the contact stress at that contact point. Zhao et al.^28,29^ used the finite element analysis method to establish a generalized fretting contact model between adjacent layers of steel strands. According to the stress characteristics of stranded wire, he analyzed the influence of the fretting parameters, such as the friction coefficient, contact stress, and shaft end tension, on the stress distribution of the contact area. According to the principle of wire rope twisting, Wang et al.^30,31^ established a geometric structure model of steel wire ropes and a boundary condition model of wire-rope torsional finite elements based on considering the spatial geometry of steel wire rope. Duan et al.^32,33^ systematically expounded the spatial geometric design theory of steel wire rope and its structural design calculation method and discussed the curvature of twisted wire, the double helix model, and the spatial geometric equations of single and double-twisted rope wire.

In summary, there have been many studies on steel strands but few on preformed helical fittings. As preformed helical fittings are widely used on overhead wires, it is becoming more and more important to carry out research to improve the performances of preformed helical fittings. The tightening characteristic and pull-out resistance of preformed helical fittings were investigated by means of experiments and finite element simulations in this study.

## Geometric mathematical model and finite element model of preformed helical fittings

### Geometric model of preformed helical fittings

**Figure 2:**
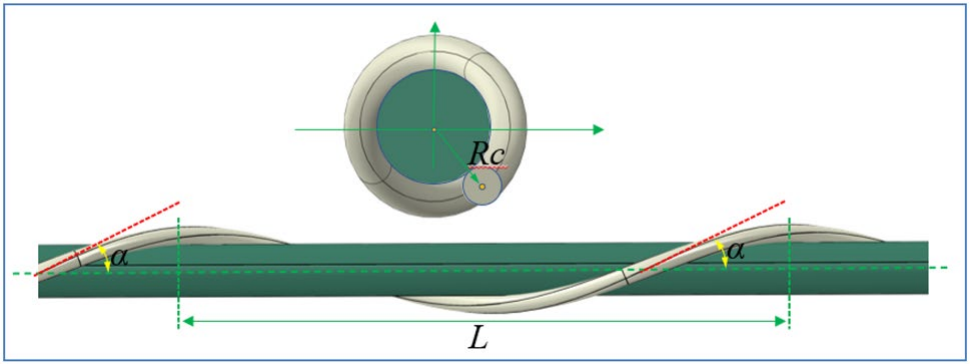
Schematic diagram of preformed armor rod winding.

Before establishing a finite element model of preformed helical fittings, it is necessary to sort out the geometric model of the preformed helical fittings to lay the foundation for subsequent parametric modeling. The geometric characteristics of the preformed armor rod are mainly reflected in the pitch, rotation direction, lay angle, and diameter. The pitch L is the axial distance of the preformed armor rods after 360° of rotation around the core. The rotation direction of the preformed armor rod product depends on the rotation direction of the outermost strand of the wire, and the rotation direction of the power fitting product will be consistent with the rotation direction of the cable, but in the optical cable fitting product, the opposite of the optical cable rotation direction will be selected in some specific cases. The lay angle α is also called a helix angle, its right rotation is positive and the left rotation is negative. The actual winding of preformed armor rods with the wire is equivalent to that shown in Fig. 2, with *R*_*c*_ being the radius of the center wire. The winding satisfies the following:

1 $$\tan \alpha = \frac{{2\pi R_{{\text{c}}} }}{L}.$$

Assuming that the angle φ in the left-handed stranded line is negative, the mathematical equation for the centerline of the pre-stranded wire is shown as follows:

2 $$\left.\begin{array}{c}x\left(\varphi \right)={R}_{j}\mathit{cos}\left({\xi }_{j}+q\varphi \right)\\ y\left(\varphi \right)={R}_{j}\mathit{sin}\left({\xi }_{j}+q\varphi \right)\\ z\left(\varphi \right)=\varphi \frac{{R}_{j}}{\mathit{tan}{\alpha }_{j}}\end{array}\right\}.$$

**Figure 3:**
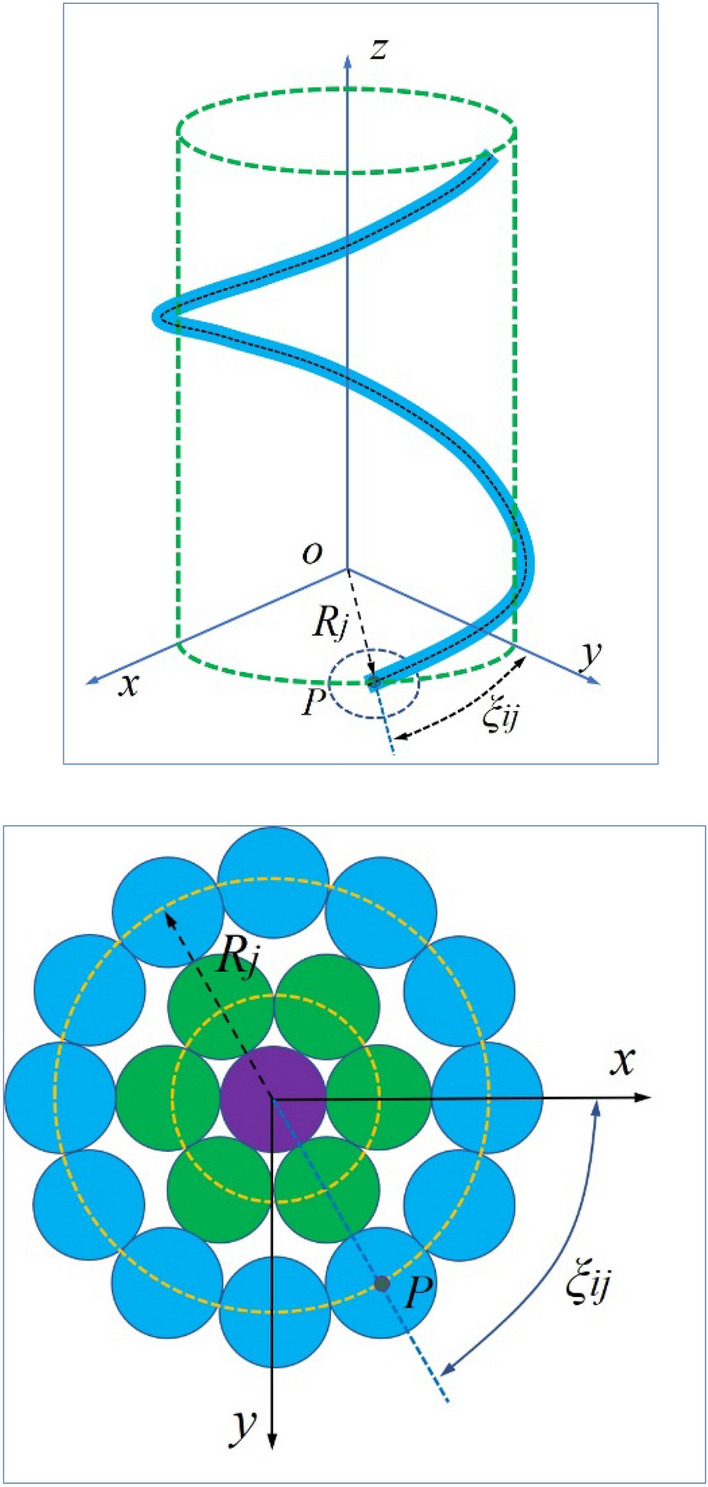
Parameter definitions at the centerline of preformed armor rods.

*q* = 1 when the winding is right-handed and *q* =  − 1 when the winding is left-handed. j represents the j-th layer of the wire wound on the central axis. i represents the *i*-th wire in the *j*-th layer of wire wound. Parametric Eqs. (2) can define the shape and position of any pre-stranded geometry on a wire, so they are sufficient to design a 3D mathematical model of the preformed armor rods. This is shown in Fig. 3.

### Finite element modeling and verification of preformed helical fittings

**Figure 4:**
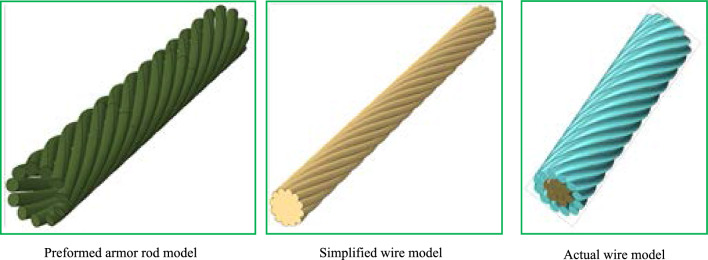
Model diagrams of preformed helical fittings considering the concave and convex characteristics of the wire.

Indeed, a transmission wire is a component of multiple non-insulated single wires stranded to transmit a current. Its surface is characterized by concave and convex characteristics. To improve the efficiency of the calculation while ensuring the accuracy of the finite element analysis, we have retained the bump and convex characteristics of the outermost preformed armor rod, ignoring the complex composition of the central core. This model only simulated the concave and convex shapes of the wire edges, simplifying the interior, as shown in Fig. 4.

To eliminate rigid body displacement and reduce the number of iterations required to calculate the contact state, the constraint of the preformed armor rods was defined as an overall common force without relative motion, so the adjacent preformed armor rods were bound sequentially. The core and preformed armor rods in the preformed helical fittings were in close contact, and finite sliding formulas of the finite slip type were used, allowing for any amount of relative movement between the contact surfaces. Because the contact area was entirely curved, there were many contact areas, and the number of surface–surface discrete calculations was very large, the discretization method was the node-surface type. The slave surface nodes did not penetrate the master surface in the calculation, while the master surface nodes could penetrate the slave surface.

**Figure 5:**
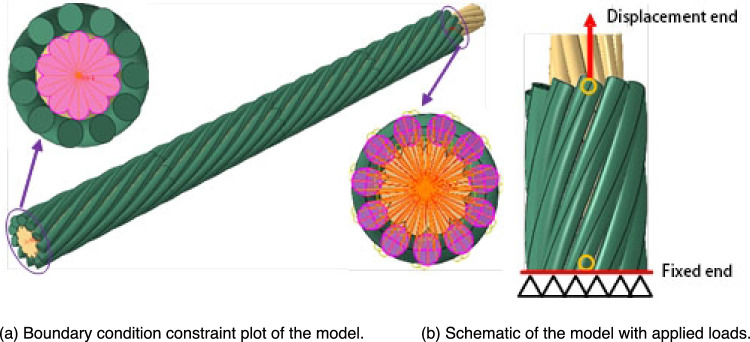
Constraint diagram of the model boundary condition and the applied load diagram under the concave and convex characteristics of the wire.

According to the actual engineering installation of the preformed helical fittings, the wire at one end close to the cardioid pull ring was fixed at the same level as that of the preformed armor rods. A reference point RP-1 was established in the center of the end face of this wire. The reference point was established with the end face through a coupling, and the other end of the wire was free. An axial tensile displacement load was applied to the preformed armor rod end face of the free end of the wire, and a new reference point RP-2 was created in the direction of the preformed armor rod axis. Coupling was also used to establish a coupling connection between this reference point and the end face of the free end of the wire, and the other end of the preformed armor rod was free. The boundary condition constraints and load settings of the model are shown in Fig. 5. When a displacement was applied at the top of the model (Fig. 5b), the displacement change was calculated by finite element software as the required tensile force. This was called the grip force, which was used to evaluate the grip performance of the preformed armor rods.

**Figure 6:**
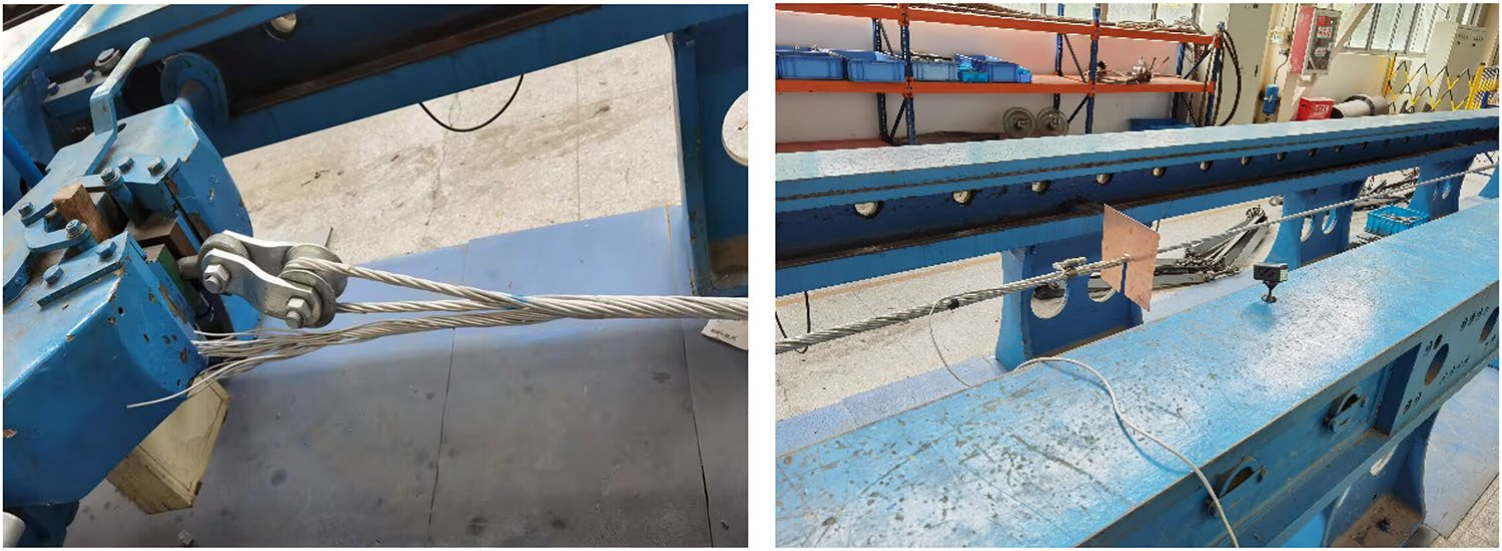
Preformed helical fitting test instruments and preformed armor rods.

To verify the rationality of the finite element method, a drawing test of the preformed armor rods was carried out. This experimental study used the ± 800-kV pre-stranded tensile wire clamp NL-150BG-20 (HZ-122007) of the State Grid Corporation of China as an example. The grounding wire was the aluminum-clad strand LBGJ-150-20AC with an outer diameter of 15.75 mm. The calculated tensile force was 178.57 kN, and the test specimen is shown in Table 1. The preformed armor rod was the YB/T123-2017 standard aluminum-clad steel wire, and the test equipment is shown in Fig. 6.

**Table 1:** Results of the pre-stranded tensile grip test.

Operating condition	Radius (mm)	Pitch (mm)	Length (mm)	Forming aperture (mm)	Pull-up resistance (kN)		
					The first group	The second group	The third group
1	2.6	180	1600	φ13.1	193.8	192.1	193.2
2		185			188.7	192.2	193.0

**Figure 7:**
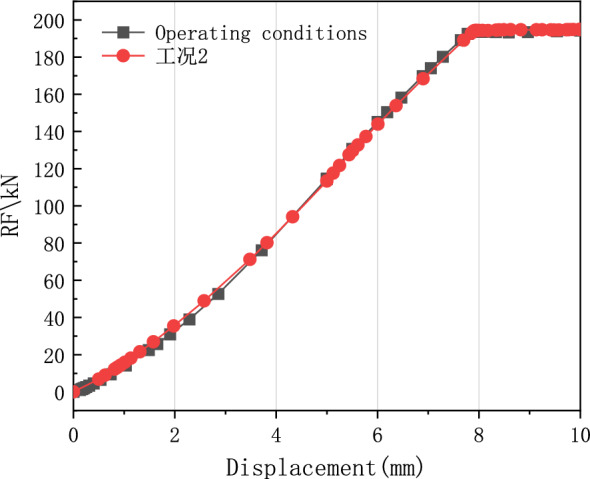
Results of the calculations of the finite element model.

According to the test parameters, the finite element model was established. The pulling force of the test is shown in Table 1. A finite element model consistent with the test was established, and the simulation results of the pulling forces obtained by the calculations are shown in Fig. 7. The comparison of results showed the calculation results of the finite element model were consistent with the experimental results.

## Finite element simulation of preformed helical fittings considering concave–convex characteristics of wire

In this section, four parameters that affect the fastening characteristics of preformed helical fittings are analyzed: the forming aperture, length, pitch, and diameter. Because the interaction mechanism between the parameters is too complex, this paper only considers the main effect of each parameter on the grip force. The interaction between the parameters is not considered.

### Influence of forming aperture on fastening characteristics of preformed helical fittings

**Figure 8:**
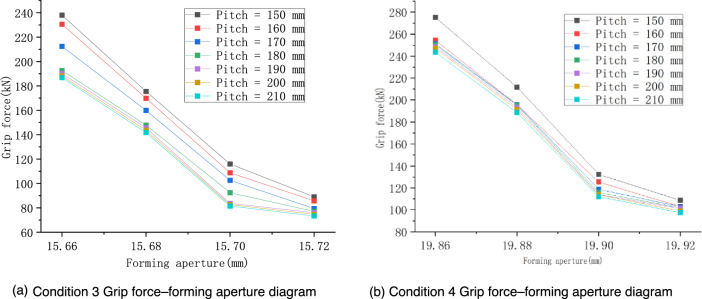
Effect of forming aperture on the fastening characteristics of preformed helical fittings under the concave and convex characteristics of the wire.

Under the condition that the other parameters remain unchanged, two groups of 56 working conditions were set to study the influence of the forming aperture on the fastening performances of preformed helical fittings, as shown in Table 2. To reflect the variations in the grip strength comprehensively, the preformed armor rod pitch also took multiple values, each time being incremented by 10 mm. The simulation results under different forming apertures are shown in Fig. 8.

**Table 2:** Specific operating conditions under the influence of the forming aperture.

Operating condition	Preformed armor rod diameter (mm)	Wire diameter (mm)		Forming aperture (mm)	Pitch of preformed armor rods (mm)
3	5.2	15.8		15.66, 15.68, 15.70, 15.72	150–210 (Increment = 10)
4	7.0	20.0		19.86, 19.88, 19.90, 19.92

From the results in Fig. 8, it can be seen that the variation trend was the same as that in the previous section. The grip force decreased with the increase in the forming aperture, and the variations of the grip force under different operating conditions were consistent, which were independent of the pitch of the preformed armor rods. In subsequent calculations, the interference amount of Δƒ = 0.1 mm was uniformly used for the simulation. Based on the simulation calculation results of the two groups of gripping forces, the interference amount was within the range of 0.08–0.12 mm, and the negative linear relationship between the gripping force and the forming aperture was more evident. In operating condition 4, when the pitch was 150 mm, the grip force changed from 275.37 kN when the forming aperture was 19.86 mm to 132.4 kN at 19.90 mm. When the interference was in the range of 0.12–0.14 mm, the change of the grip force with the forming aperture was slow, especially in the case of a large pitch (190–210 mm), which means that increasing the pitch at this time weakened the effect of the forming aperture on the grip force.

From the comparison of the grip force results in Fig. 8, it was found that the grip force increased with the decrease in the forming aperture. However, for convenience of installation, the forming aperture of the preformed armor rods should not be too small. In addition, if the forming aperture of the preformed armor rods were too small, it could easily wear the core. Therefore, it is very important to choose a suitable forming aperture size considering various factors.

### Influence of length on fastening characteristics of preformed helical fittings

Considering the concave and convex characteristics of the wire, in this section, we calculate and analyze the effect of the length on the fastening performance of preformed helical fittings. The operating conditions are shown in Table 3, which were labelled operating conditions 5 and 6, with a total of 72 working conditions.

**Table 3:** Specific operating conditions were set under the influence of the length of the preformed armor rods.

Operating condition	Preformed armor rod diameter (mm)	Core diameter (mm)	Pitch numbers	Pitch (mm)
5	5.2	15.8	3, 4, 5, 6, 7, 8	150–200 (Increment = 10)
6	7.0	20.0	3, 4, 5, 6, 7, 8	

**Figure 9:**
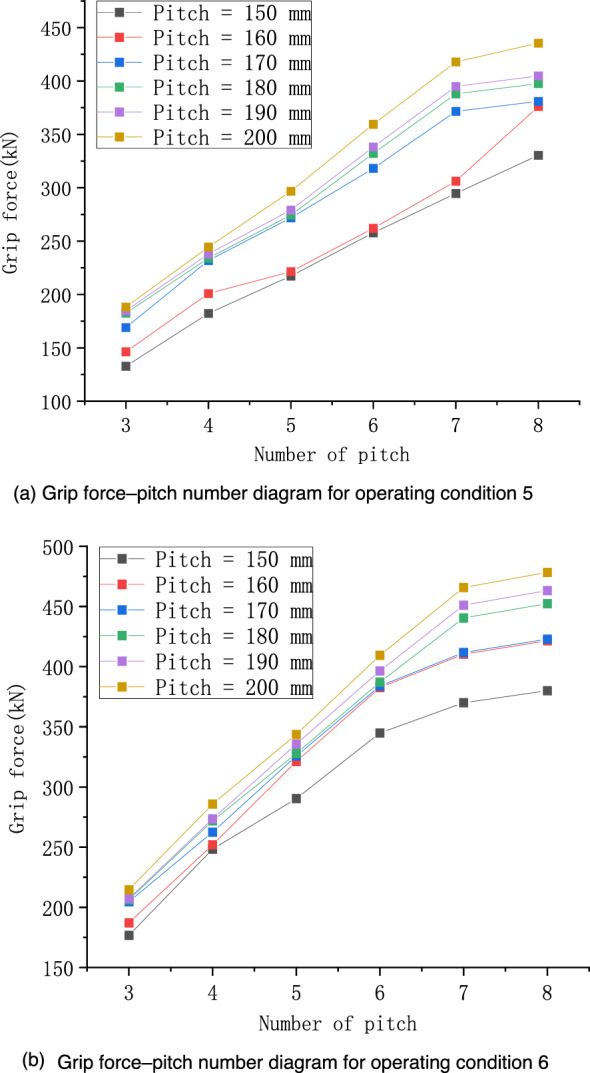
Relationship diagram of grip force–pitch number of preformed helical fittings under various working conditions.

From Fig. 9, it can be seen that the grip force increased with the increase in the length, and from the simulation results of operating conditions 5 and 6, the grip force of the preformed armor rods increased significantly in the pitch number range of 3–7. When the pitch number of the preformed armor rods was 7 or 8, the grip force changed less with the length, indicating that when the length was increased to a certain extent, the increase in length would not play a role in increasing the tightening performance. Therefore, in practical applications, the length of the preformed armor rods should not be too short; otherwise, the grip force will be insufficient. However, it should not be too long, because after the preformed armor rods reaches a critical length, increasing the length will not increase the grip force but will only cause an increase in cost.

**Figure 10:**
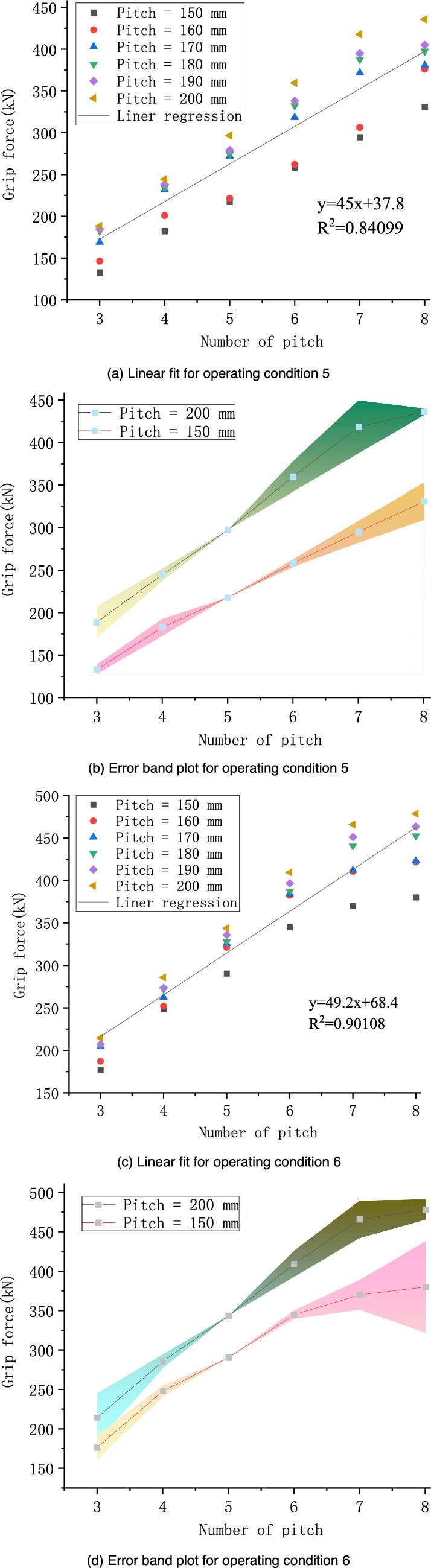
Linear fitting and error analysis of preformed helical fittings under various working conditions.

The relationships between the grip force and the preformed armor rod pitch number under two sets of operating conditions were linearly fitted. The fitted lines were translated to the position where the number of pitches was 5, and the pitches were 150 and 200 mm. Error analysis was performed, as shown in Fig. 10.

The slopes of the fitted lines for conditions 5 and 6 were 45 and 49.2, respectively. Calculated from the error band plot, the maximum relative errors of the grip force of working condition 5 for pitches of 150 and 200 mm were 6.60% and 9.83%, respectively. The maximum relative errors of the grip force for pitch operating condition 6 were 15.26% and 14.31% when the pitch was 150 and 200 mm, respectively. The relative errors of these grip force are within 12%, which has certain reference value.

### Effect of preformed armor rod pitch on fastening characteristics of preformed helical fittings

**Figure 11:**
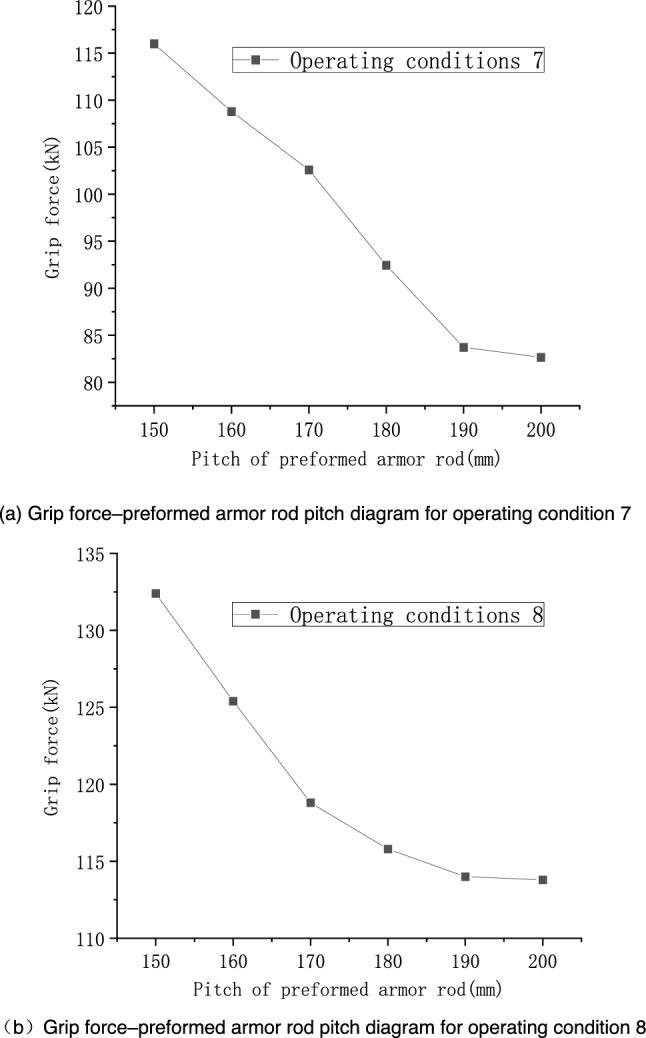
Relationship of grip force and stress with preformed armor rod pitch under various operating conditions.

**Figure 12:**
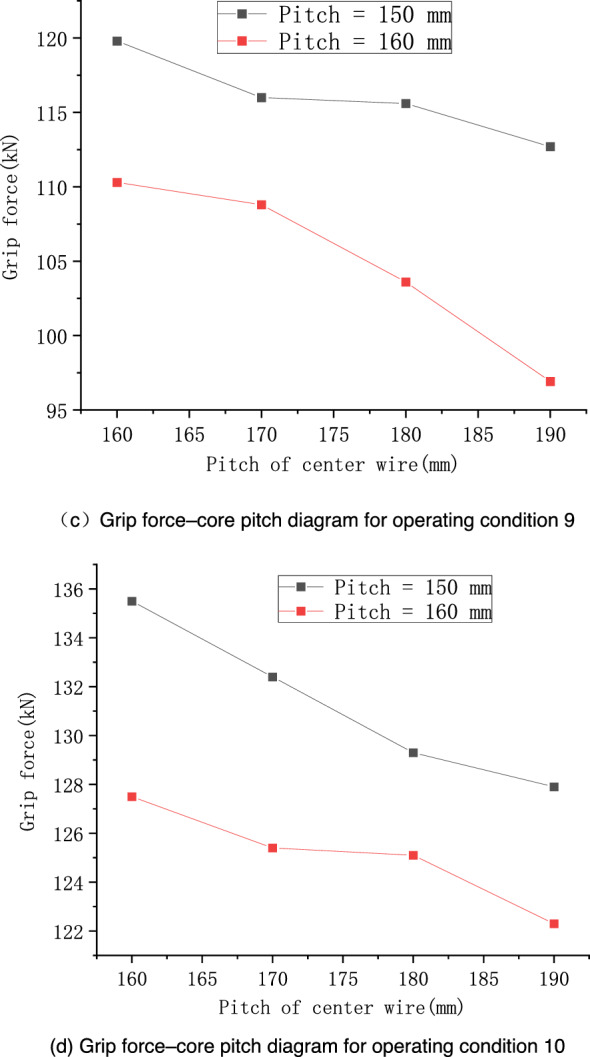
Relationship of grip force and stress with core pitch under various operating conditions.

When considering the concave and convex surfaces of the wire, the wire has stranding characteristics. To perform a comprehensive analysis, the influences of the preformed armor rod pitch and the core pitch on the results were studied separately. A section with 320-mm-long fittings was considered, and the simulated operating conditions are shown in Tables 4 and 5. Finally, operating conditions 7 and 8 were specified. To fully reflect the variation trends of the grip force, Table 4 shows two sets of preformed armor rod pitches. The stress cloud diagram of the seventh operating condition is shown in Fig. 14, and the results of each operating condition are shown in Figs. 11 and 12.

**Table 4:** Specific operating conditions under the influence of the preformed armor rod pitch.

Operating condition	Preformed armor rod diameter (mm)		Core diameter (mm)	Preformed armor rods /size of core mesh (mm)	Pitch of core (mm)	Pitch of preformed armor rods (mm)
7	5.2		15.8	1.0/2.0	170	150–200 (Increment = 10)
8	7.0		20.0

**Table 5:** Specific operating conditions set under the influence of core pitch.

Operating condition	Preformed armor rod diameter (mm)	Core diameter (mm)	Size of reformed armor rod/core mesh (mm)	Pitch of core (mm)	Pitch of preformed armor rods (mm)
9	5.2	15.8	1.0/2.0	150	160–190 (Increment = 10)
10	7.0	20.0		160

**Figure 13:**
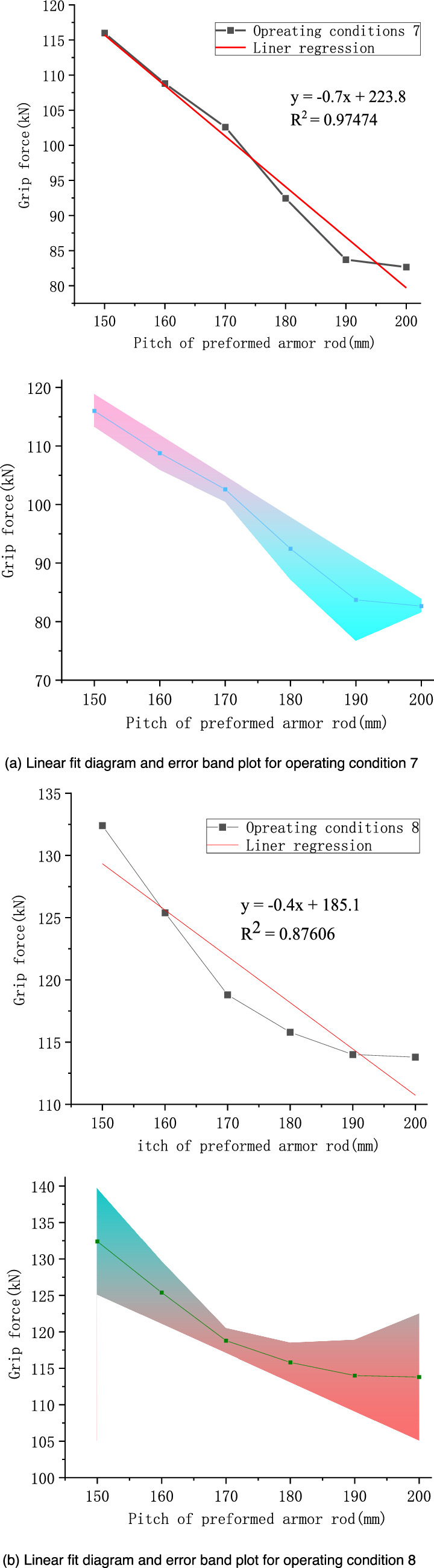
Linear fitting and error analysis of preformed helical fittings under various operating conditions.

According to Fig. 11, the grip force decreased with the increase in the preformed armor rod pitch. From the simulation results of operating conditions 7 and 8, it can be seen that when the preformed armor rod pitch was in the range of 150–190 mm, the grip force decreased significantly. In particular, the grip force for operating conditions 7 at the pitch of 150 mm was 116.0 kN, and the grip force at the pitch of 190 mm was 83.71 kN, which basically showed a linear downward trend, and the decline rate was roughly 0.81 kN/mm. When the preformed armor rod pitch was in the range of 190–200 mm, the grip force and stress were basically the same, and the change was slow, indicating that when the preformed armor rod pitch increased on the basis of a pitch of 200 mm, there was no obvious impact on its fastening performance. There was a negative correlation between the pitch of the core and the grip force of the preformed helical fittings. When the core pitch was in the range of 160–190 mm, the change in the grip force and stress was gentler than when the preformed armor rod pitch changed, indicating that the factor affecting the grip force was the preformed armor rod pitch, and the change of the core pitch can be ignored in general. The values of the grip force as the pitch varied under the two sets of working conditions were linearly fitted, and error analysis was performed, as shown in Fig. 13.

From Fig. 13, it can be seen that the slopes of the fitted lines for conditions 7 and 8 were − 0.7 and − 0.4, respectively. From the error band plot, the maximum relative error of the grip force in operating condition 7 at a pitch of 190 mm was 8.47%, and the maximum relative error of the grip force at a pitch of 200 mm in operating condition 8 was 7.64%. Thus, the slope could predict the grip force value accurately.

**Figure 14:**
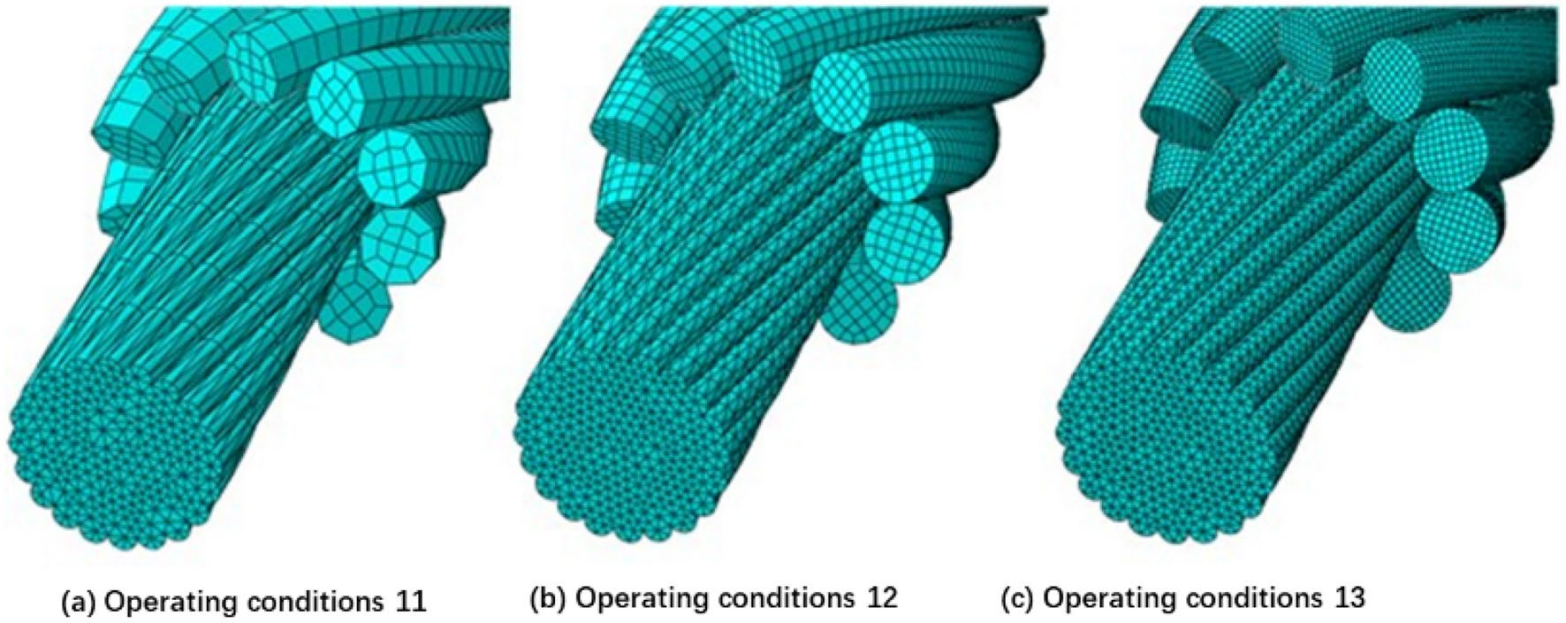
Mesh at different sizes.

As shown in Table 6, three operating conditions with different pitches and different mesh sizes were set to investigate the contact status between the preformed armor rod and the core under different conditions. Moreover, this test will help select suitable mesh sizes in the analysis of finite element simulation. The radius of the preformed armor rod was 2.6 mm, the length was 160 mm, and the pitch was 120–220 mm. The radius of the core was 6.7 mm and the pitch was 180 mm. The preformed armor rod rotated in the same direction as the core (right-handed). The preformed armor rod mesh was C3D8R, and the core mesh was C3D10. The parameter settings and interactions in the model were consistent with the previous model, and mesh sensitivity analysis was performed on this model. Models with different mesh sizes are shown in Fig. 14.

**Table 6:** Mesh sensitivity analysis of case parameters considering wire surface effects.

Operating condition	Mesh size of preformed armor rod	Mesh size of core
11	2.0	6.0
12	1.0	2.0
13	0.5	1.0

**Figure 15:**
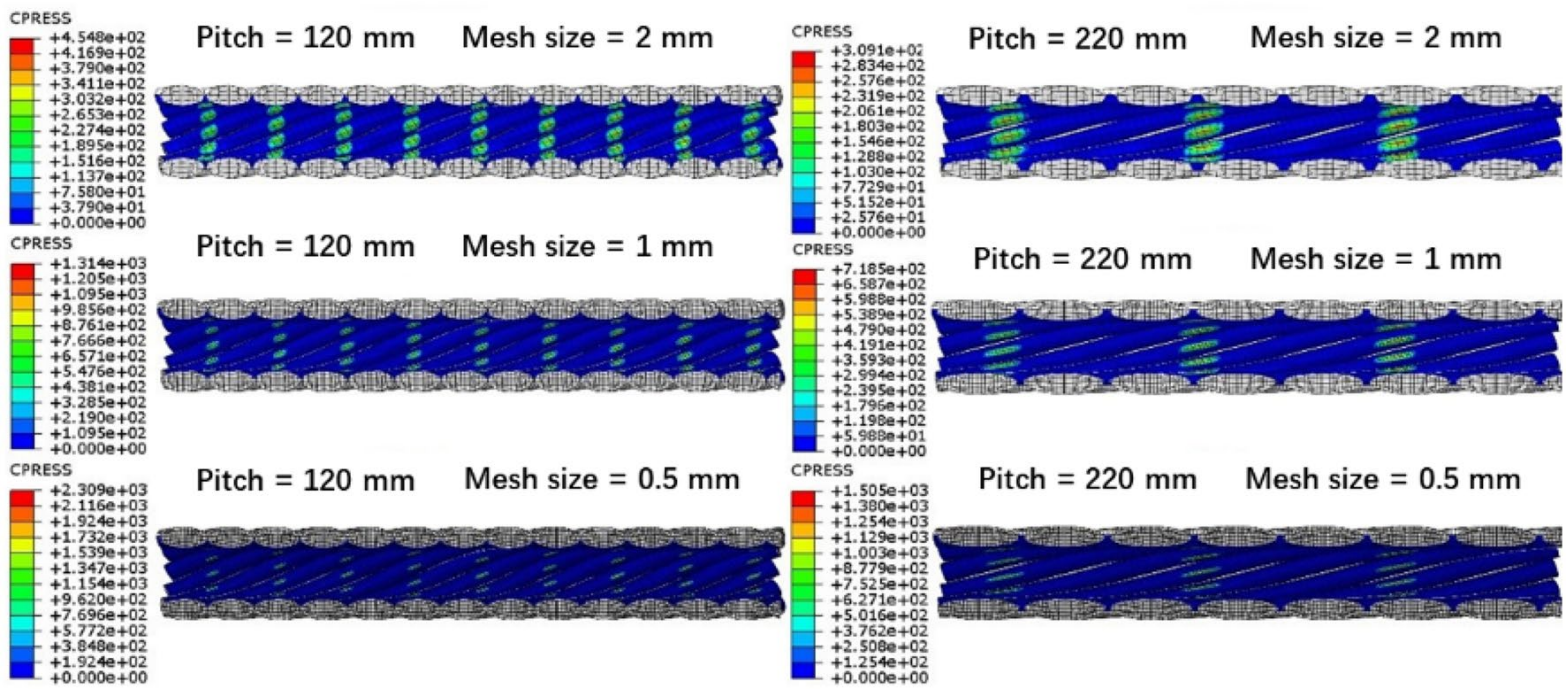
Compressive stress between preformed armor rods and core under different mesh sizes.

Figure 15 shows the normal contact stress between the preformed armor rod and the core, and the calculation results showed that the contact points at the 120-mm pitch significantly increased compared to those at the 220-mm pitch under the same grid size, and the contact stress was greater. At the same time, with increasing mesh density, the contact points increased; thus, the CPRESS between the preformed armor rod and the core gradually increased, improving the contact state between the two. This section mainly analyzes the influence of preformed armor rod parameters on the anti-pulling force, so the selection of the mesh size should be appropriate, which can not only ensure the calculation accuracy but also improve the calculation efficiency.

### Effect of preformed armor rod diameter on fastening characteristics of preformed helical fittings

**Figure 16:**
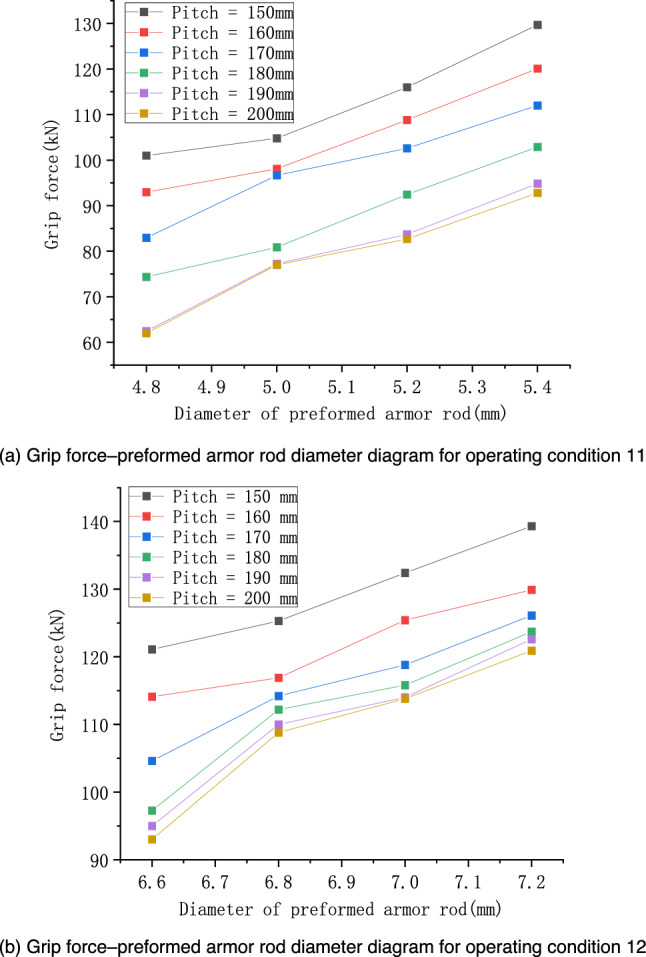
Relationship of grip force and diameter considering the concave and convex characteristics of the wire.

In this subsection, the effect of the preformed armor rod diameter on the fastening properties of the preformed helical fittings is analyzed, taking into account the concave and convex characteristics of the wires. The operating conditions are shown in Table 7, and these two cases were named operating conditions 14 and 15. All the other parameters were unchanged, and the results are shown in Fig. 16.

**Table 7:** Specific operating conditions under the influence of preformed armor rod diameter.

Operating condition	Preformed armor rod diameter (mm)	Core diameter (mm)	Diameter of preformed armor rods (mm)	Pitch of preformed armor rods (mm)
14	5.2	15.8	4.8–5.4 (Increment = 0.2)	150–200 (Increment = 10)
15	7.0	20.0	6.6–7.2 (Increment = 0.2)

From the calculation results of Fig. 15, it can be seen that the grip force of the preformed helical fittings increased with the increase in the diameter of the preformed armor rods. The smaller the pitch value, the more significant the effect of increasing the diameter of the preformed armor rods on the increase in the grip force. For example, at a pitch of 150–180 mm, the grip force increased quickly with the diameter of the preformed armor rod. For example, when the preformed armor rod pitch was 150–180 mm, the grip force value gradually flattened with the change in the preformed armor rod diameter. When the preformed armor rod pitch was 190 or 200 mm, increasing the preformed armor rod diameter had little effect on the grip force value. This showed that the effect of the change in the pitch of the preformed armor rods on the grip force was greater than the effect of the change in the diameter of the preformed armor rods, which weakened the influence of the diameter on the result.

**Figure 17:**
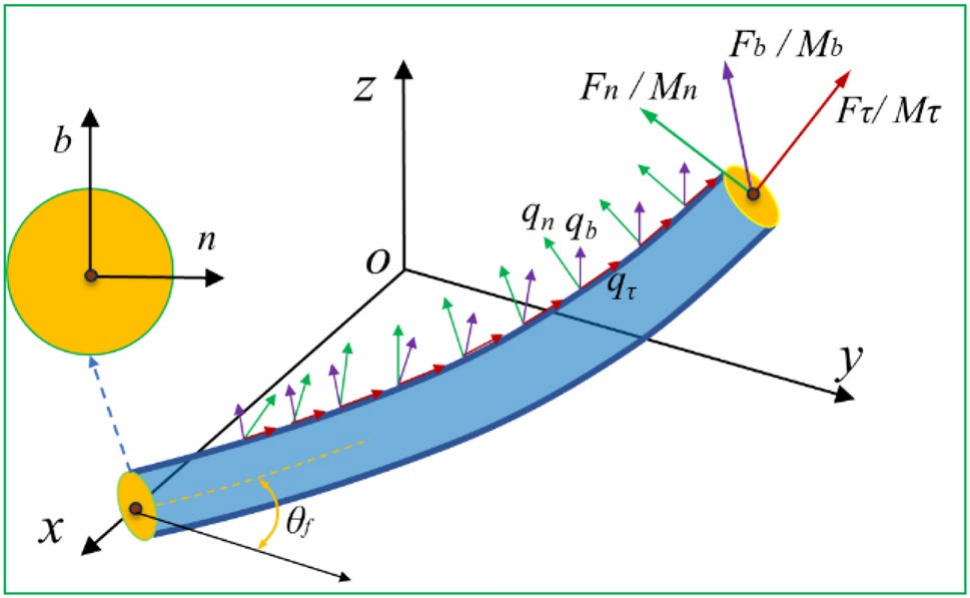
Mechanical model of preformed armor rods.

In addition, in order to further analysis the influence of the diameter of the preformed armor rods on the grip force, we can make a theoretical analysis from the direction of Castro’s theory. Firstly, we establish the geometric model of the preformed armor rods model. This model is composed of preformed armor rods with large stiffness. In order to describe the geometric characteristics of the preformed armor rods and ignore the structure of the outer surface of the core. Take the segment model of the preformed armor rods, as shown in Fig. 17. The interaction forces between the preformed armor rods and the core are *q*_n_, *q*_b_ and* q*_τ_. Under the action of external force, there are internal forces F_n,_ F_b_ and F_τ_ on the cross section of the preformed armor rods, as well as internal moments M_n_, M_b_ and M_τ_ in three directions.

According to the mechanical model, the micro-element is divided, and the force balance equation of the preformed armor rods can be established:

3 $$\begin{aligned} & \frac{{{\text{d}}F_{\tau } }}{{{\text{d}}s}} - \kappa_{b} F_{n} + \kappa_{n} F_{b} + q_{\tau } = 0{ ;}\;\frac{{{\text{d}}M_{\tau } }}{{{\text{d}}s}} - \kappa_{b} M_{n} + \kappa_{n} M_{b} = 0 \\ & \frac{{{\text{d}}F_{n} }}{{{\text{d}}s}} - k_{\tau } F_{b} + \kappa_{b} F_{\tau } + q_{n} = 0{;}\;\frac{{{\text{d}}M_{n} }}{{{\text{d}}s}} - k_{\tau } M_{b} + \kappa_{b} M_{\tau } - F_{b} = 0 \\ & \frac{{{\text{d}}F_{b} }}{{{\text{d}}s}} - \kappa_{n} F_{\tau } + k_{\tau } F_{n} + q_{b} = 0{;}\;\frac{{{\text{d}}M_{b} }}{{{\text{d}}s}} - \kappa_{n} M_{\tau } + k_{\tau } M_{n} + F_{n} = 0 \\ \end{aligned}$$

In Eq. (3), s is the winding length of the preformed armor rod, k_n_, k_b_ and k_τ_ describe the rotation speed of the natural coordinate system with s, respectively. Equation (3) is the equilibrium equation for calculating the statics of the preformed armor rods. There are 12 unknown quantities, 6 internal forces, 3 curvatures and 3 external distributed loads in the equation. If the initial twist angle and the outer diameter of the core are known, three curvatures k_n_, k_b_ and k_τ_ can be calculated. According to Fig. 17, it can be seen that if the initial curvature and the deformed curvature are determined, the relationship between curvature and internal torque can be obtained according to the properties of the cross section. According to the axial inertia moment and polar inertia moment of the n-axis and b-axis of the circular section, the relationship between curvature and internal moment can be obtained.

4 $$M_{\tau } = \frac{{\pi r^{4} }}{4(1 + \mu )}E{(}k_{\tau } - k_{\tau }^{0} {) ;}M_{b} = \frac{{\pi r^{4} }}{4}E{(}k_{b} - k_{b}^{0} {);}M_{n} = 0$$

According to Eq. (4), the internal torque can be obtained, and the number of unknowns in Eq. (3) will be reduced to 6. Because it is difficult to solve Eq. (3), it needs to be solved iteratively, and numerical methods are generally used. In order to obtain a formula with guiding value and analyze the fastening mechanism of preformed armor rods, some simplified assumptions are made in this paper. It is considered that the curvature of the preformed armor rods is constant along the arc length and the curvature in the direction of n is zero during the tension process. Based on this assumption, the following simplified equation can be obtained.

5 $$\begin{aligned} \frac{{{\text{d}}F_{\tau } }}{{{\text{d}}s}} + q_{\tau } & = 0{; }\frac{{{\text{d}}M_{\tau } }}{{{\text{d}}s}} = 0 \\ - k_{\tau } F_{b} + \kappa_{b} F_{\tau } + q_{n} & = 0{; } - k_{\tau } M_{b} + \kappa_{b} M_{\tau } - F_{b} = 0 \\ q_{b} & = 0{ ; }F_{n} = 0 \\ \end{aligned}$$

The following equation can be obtained by simplifying Eq. (3).

6 $$q_{n} = k_{\tau } \kappa_{b} M_{\tau } - k_{\tau }^{2} M_{b} - \kappa_{b} F_{\tau }$$

Equation (6) represents the general expression of the grip force q_n_ of the preformed armor rods and the core. The preformed armor rods can be firmly wound on the core to resist the slip force, which is due to the friction provided by the grip force q_n_. If the tension of the preformed armor rods exceeds the maximum static friction provided by the grip force q_n_, the preformed armor rods begin to slide. The Eq. (6) shows that increasing M_b_ and F_τ_ can increase the grip force between the core and the preformed armor rods. The greater the bending moment along the sub-normal direction, the greater the force of the preformed armor rods to resist slip, and the stronger the grip force. Substituting Eq. (3) into Eq. (6), we can get the following equation.

7 $$q_{n} = \frac{{\pi r^{4} }}{4(1 + \mu )}Ek_{\tau } \kappa_{b} {(}k_{\tau } { - }k_{\tau }^{0} {)} - \frac{{\pi r^{4} }}{4}E{(}k_{b} { - }k_{b}^{0} {)}k_{\tau }^{2} - \kappa_{b} F_{\tau }$$

From the Eq. (7), it can be concluded that the grip force of the preformed armor rods is linearly proportional to the fourth power of the radius of the preformed armor rods.

**Figure 18:**
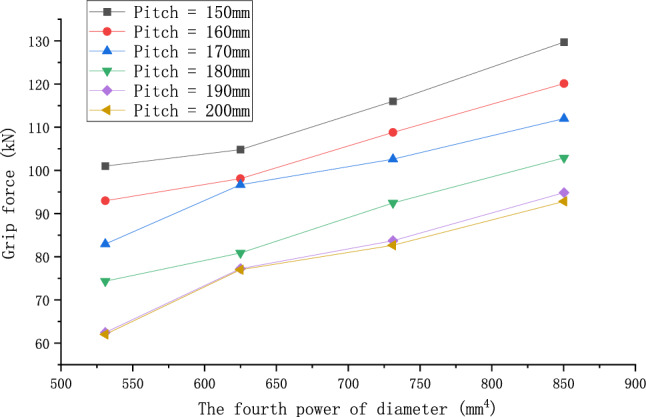
Relationship of grip force and the fourth power of its diameter considering the concave and convex characteristics of the wire.

**Figure 19:**
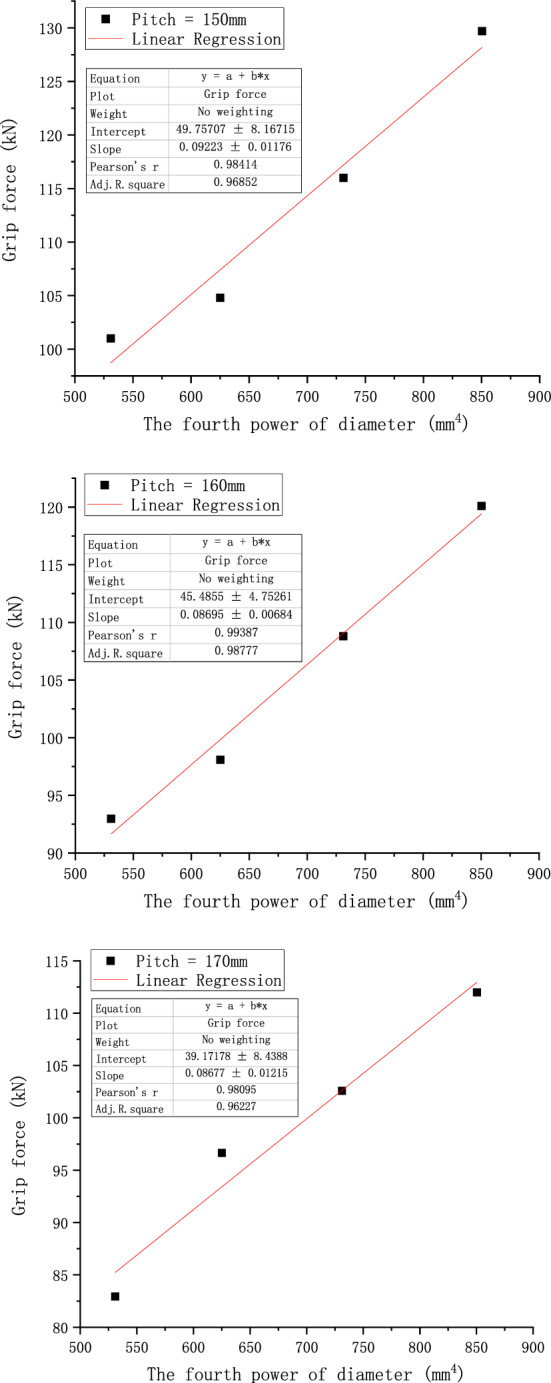

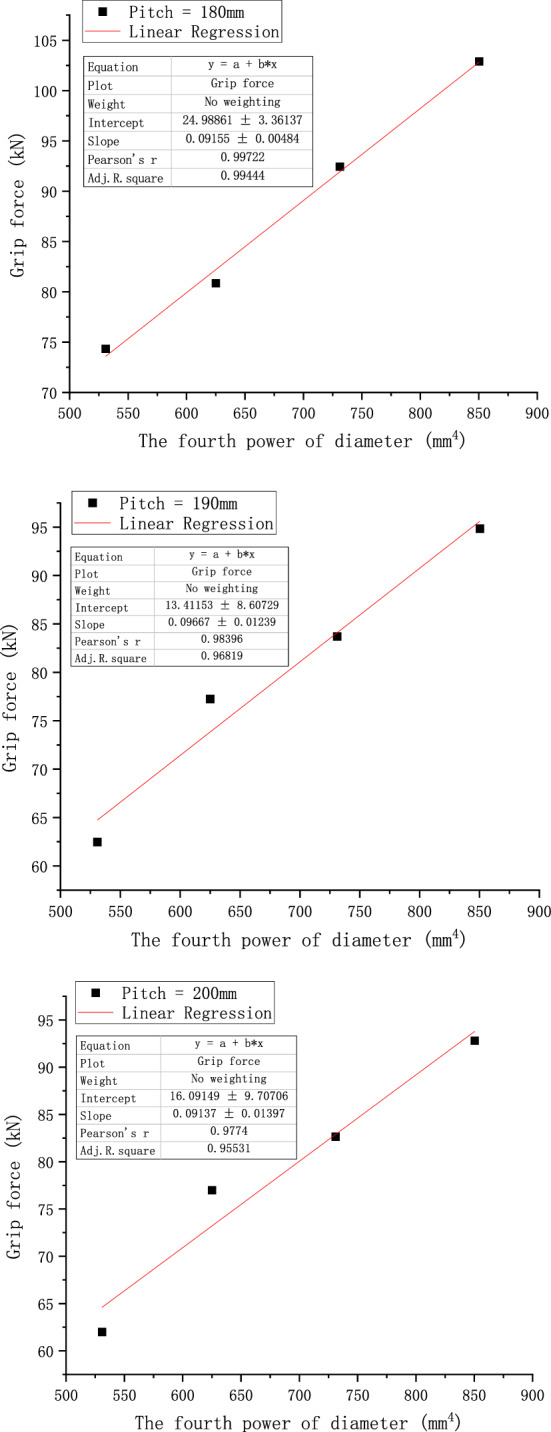
Linear fitting curve of each pitch graph.

According to the conclusions derived from the above theoretical derivation, we analyze Fig. 16a in the paper and process its data. We replace the diameter in the graph with the fourth power of the diameter, as shown in Fig. 18, but it may be difficult to tell the difference from Fig. 18 because of the scale problem. Therefore, we do linear fitting of the curves under different pitches, and get Fig. 19. From Fig. 19, we can get the value of R^2^ of linear fitting of each curve, as shown in Table 8 below.

**Table 8:** The value of R^2^ under each pitch.

Pitch (mm)	150	160	170	180	190	200
R^2^	0.96852	0.98777	0.96227	0.99444	0.96819	0.95531

It can be seen from the above table that the values of R^2^ are very close to 1, indicating that the effect of linear fitting is good, which indicates that there is a certain linear relationship between the grip force of the preformed armor rods and the fourth power of its diameter.

After completing the theoretical analysis and finite element data analysis, we also did a simple single-wire scale experiment to verify that there is a linear relationship between the fourth power of the diameter of the preformed armor rods and the grip force.

**Figure 20:**
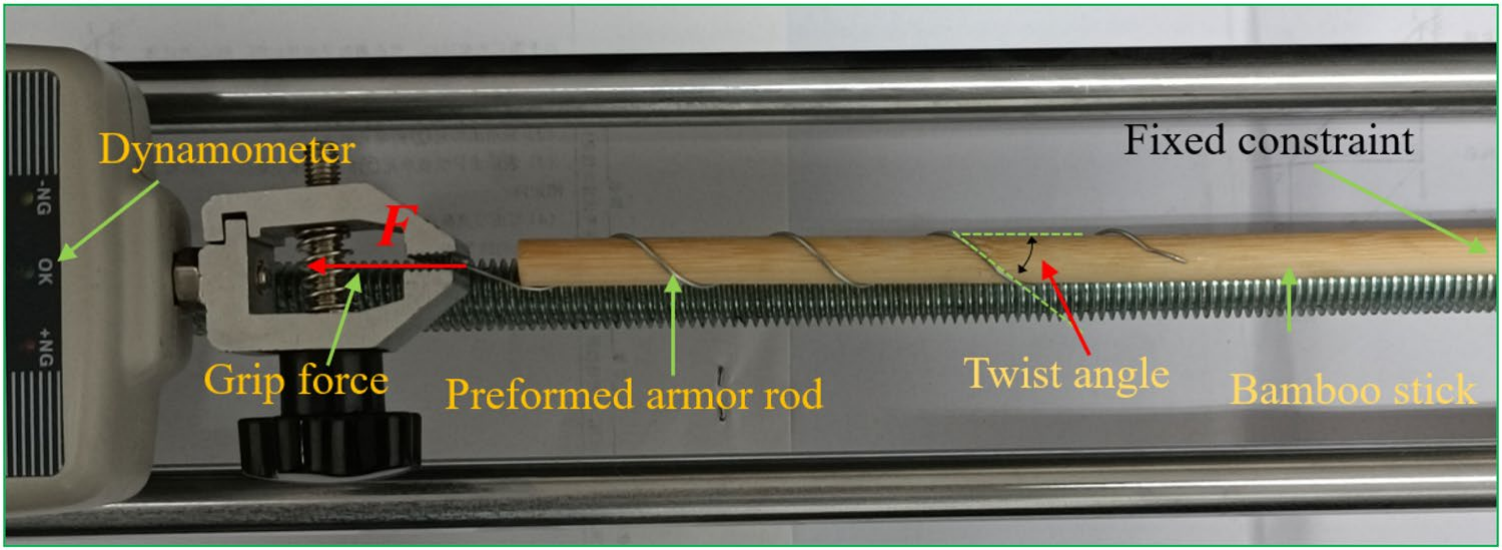
The diagram of the experiment.

The experimental instrument used in this scale experiment is Aigu electronic digital push–pull meter, with a total length of 680 mm. The experimental material is 65 MN steel spring. 65 MN wire diameter is 0.4 mm, 0.6 mm, 0.8 mm. The outer diameter of the spring is 6–10 mm. The length of the bamboo stick is 30 cm, and the outer diameters are 6 mm, 7 mm, 8 mm. In this experiment, the production process of preformed armor rods is similar to that of hard extrusion forming spiral wire, because the spring is wound on the object, which is equivalent to having its own inward binding force. When stretching at both ends, it can form a similar pre-twist structure with inward prestress. This can be used to study the fastening characteristics of preformed helical fittings. The diagram of the experiment is shown in Fig. 20.

**Figure 21:**
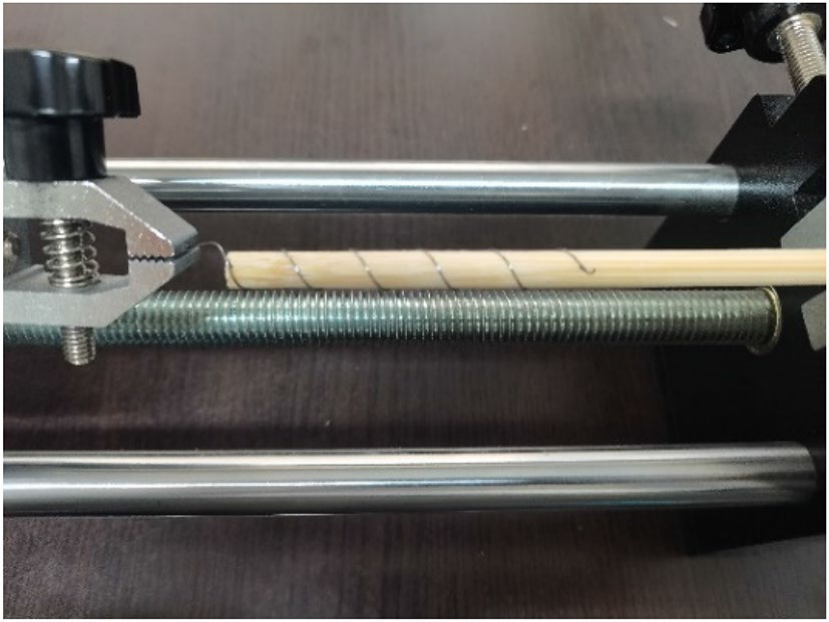
The loading diagram of the experiment.

In Fig. 21, the right end of the bamboo rod is fixed, and the spring is clamped with the chuck, and the chuck is slowly moved to the left. The tension value of the push–pull meter is read according to the slip of the right end of the spring. The spring material is No. 65 manganese steel. The spring diameter, bamboo stick diameter, pitch number and twist angle parameters are shown in Table 9.

**Table 9:** Single wire operating condition.

Operating condition	The diameter of preformed armor rods(mm)	The outer diameter of preformed armor rods(mm)	The outer diameter of bamboo stick(mm)	Initial number of pitches	Initial average twist angle (°)
1	0.6	8	7	4	37.09
2	0.4	8	7	4	38.18
3	0.8	10	10	3	29.90
4	0.6	10	10	3	30.36
5	0.8	9	8	3	28.74
6	0.6	9	8	3	29.30
7	0.8	9	8	4	28.61
8	0.6	9	8	4	29.17

In Table 9, each group of operating condition is tested in 3 groups, and the tension of the spring slip is read by the push–pull meter. A total of 24 sets of data are done, and the specific results are shown in Table 10.

**Table 10:** Comparative analysis of slippage force under various operating conditions.

Operating condition	1	2	3	4	5	6	7	8
Grip force (N)	49.97	8.00	87.57	23.73	80.07	25.74	90.27	26.17
Grip force (N)	51.82	9.62	78.95	31.95	109.0	24.10	104.00	34.85
Grip force (N)	50.21	8.73	87.68	26.02	86.59	26.17	89.96	38.44
Average grip force (N)	50.67	8.78	84.73	27.23	91.89	25.34	94.74	33.15

Due to the existence of errors, the three groups of grip forces of each operating condition are averaged before data analysis. The ratio of the grip force is obtained by dividing the grip force measured by the two operating conditions with different diameters and the same other parameters. The ratio of the fourth power of the spring diameter is calculated, and the specific value is shown in Table 11. It can be seen from Table 11 that the ratio of the grip force is close to the ratio of the fourth power of the spring diameter, and the maximum error rate is 12.85%, and the minimum is only 1.57%. This shows that Eq. (7) can reasonably reflect the mechanical mechanism of grip force.

**Table 11:** Numerical comparison results.

Operating condition	The ratio of force	The ratio of the fourth power of preformed armor rod diameter	Error rate (%)
1/2	5.77	5.06	12.28
3/4	3.11	3.16	1.57
5/6	3.63	3.16	12.85
7/8	2.86	3.16	10.59

## Conclusion

Considering the concave and convex surfaces of the transmission wires, the simplified internal structure of the wires in the ABAQUS finite element software only simulated the concave and convex characteristics of the edges. The effects of factors such as the forming aperture, length, preformed armor rod diameter, preformed armor rod pitch on the grip force were studied.

The grip force decreased with the increase in the forming aperture under different operating conditions. When the interference was 0.08–0.12 mm, a negative linear relationship between the grip force and the forming aperture was evident. When the interference amount ranged from 0.12 to 0.14 mm, the grip force changed slowly with the forming aperture, and increasing the pitch could weaken the influence of the forming aperture on the grip force. The grip force increased with the increase in the preformed armor rod length. When the pitch number of the preformed armor rods was within 3–7, the grip force increased significantly with the length of the preformed armor rods, and the grip force changed slowly with the length of the preformed armor rods at pitch numbers of 7 and 8. For the pitch, the grip force decreased with the increase in the pitch under different operating conditions. In the range of 150–180 mm, the grip force decreases significantly, and when the pitch was 190–200 mm, the grip force was basically flat. Similarly, for different operating conditions, the grip force of the preformed helical fittings increased with the increase in the diameter of the preformed armor rods. When the pitch was within 190 mm, the impact on the grip force was significant when the diameter increased, but when the pitch was greater than 190 mm, the influence of the pitch weakened the influence of the diameter on the grip force. Moreover, through the analysis of experimental, theoretical, and finite element simulation, we find that the grip force is linearly related to the fourth power of the diameter of the preformed armor rods.

## Data Availability

The datasets generated and analyzed during the current study are not publicly available due to this project is a cooperative type project but are available from the corresponding author on reasonable request.
